# 5-Meth­oxy-1-[(5-meth­oxy-1*H*-indol-2-yl)meth­yl]-1*H*-indole

**DOI:** 10.1107/S1600536812009257

**Published:** 2012-03-07

**Authors:** Mohamed I. Attia, Nasser R. El-Brollosy, Hazem A. Ghabbour, Suhana Arshad, Hoong-Kun Fun

**Affiliations:** a Department of Pharmaceutical Chemistry, College of Pharmacy, King Saud University, Riyadh 11451, Saudi Arabia; b X-ray Crystallography Unit, School of Physics, Universiti Sains Malaysia, 11800 USM, Penang, Malaysia

## Abstract

In the title compound, C_19_H_18_N_2_O_2_, the two indole ring systems are essentially planar [maximum deviation = 0.015 (2) Å in both indole ring systems] and make a dihedral angle of 72.17 (7)° with each other. In the crystal, the mol­ecules are linked into a zigzag chain along the *a* axis *via* N—H⋯O hydrogen bonds.

## Related literature

For the biological activity of melatonin (MLT), see: Csernus & Mess (2003 ▶); Nosjean *et al.* (2000 ▶); Blask *et al.* (2002 ▶); Genovese *et al.* (2005 ▶); Mills *et al.* (2005 ▶); Peres (2005 ▶); Sofic *et al.* (2005 ▶); Witt-Enderby *et al.* (2006 ▶). For related structures, see: Narayanan *et al.* (2011 ▶); Deng *et al.* (2011 ▶). For the synthesis, see: Attia *et al.* (2008 ▶).
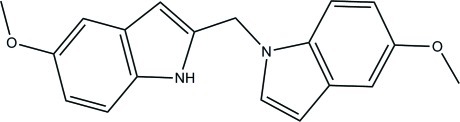


## Experimental

### 

#### Crystal data

C_19_H_18_N_2_O_2_
*M* *_r_* = 306.35
Monoclinic, 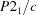

*a* = 9.4446 (5) Å
*b* = 19.5625 (8) Å
*c* = 8.6657 (5) Å
β = 98.903 (4)°
*V* = 1581.78 (14) Å^3^
*Z* = 4
Cu *K*α radiation
μ = 0.68 mm^−1^
*T* = 296 K
0.92 × 0.20 × 0.06 mm

#### Data collection

Bruker APEXII CCD diffractometer
Absorption correction: multi-scan (*SADABS*; Bruker, 2009 ▶) *T*_min_ = 0.575, *T*_max_ = 0.961
9421 measured reflections
2584 independent reflections
2087 reflections with *I* > 2σ(*I*)
*R*_int_ = 0.041

#### Refinement

*R*[*F* ^2^ > 2σ(*F* ^2^)] = 0.046
*wR*(*F* ^2^) = 0.133
*S* = 1.06
2584 reflections
215 parameters
H atoms treated by a mixture of independent and constrained refinement
Δρ_max_ = 0.15 e Å^−3^
Δρ_min_ = −0.14 e Å^−3^

### 

Data collection: *APEX2* (Bruker, 2009 ▶); cell refinement: *SAINT* (Bruker, 2009 ▶); data reduction: *SAINT*; program(s) used to solve structure: *SHELXTL* (Sheldrick, 2008 ▶); program(s) used to refine structure: *SHELXTL*; molecular graphics: *SHELXTL*; software used to prepare material for publication: *SHELXTL* and *PLATON* (Spek, 2009 ▶).

## Supplementary Material

Crystal structure: contains datablock(s) global, I. DOI: 10.1107/S1600536812009257/is5084sup1.cif

Structure factors: contains datablock(s) I. DOI: 10.1107/S1600536812009257/is5084Isup2.hkl

Supplementary material file. DOI: 10.1107/S1600536812009257/is5084Isup3.cml

Additional supplementary materials:  crystallographic information; 3D view; checkCIF report

## Figures and Tables

**Table 1:** Hydrogen-bond geometry (Å, °)

*D*—H⋯*A*	*D*—H	H⋯*A*	*D*⋯*A*	*D*—H⋯*A*
N2—H1*N*2⋯O1^i^	0.88 (2)	2.24 (3)	3.037 (2)	151 (2)

Symmetry code: (i) 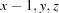.

**Fig. 1.**
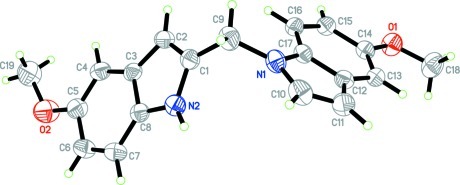
The molecular structure of the title compound, showing 30% probability displacement ellipsoids and the atom-numbering scheme.

**Fig. 2.**
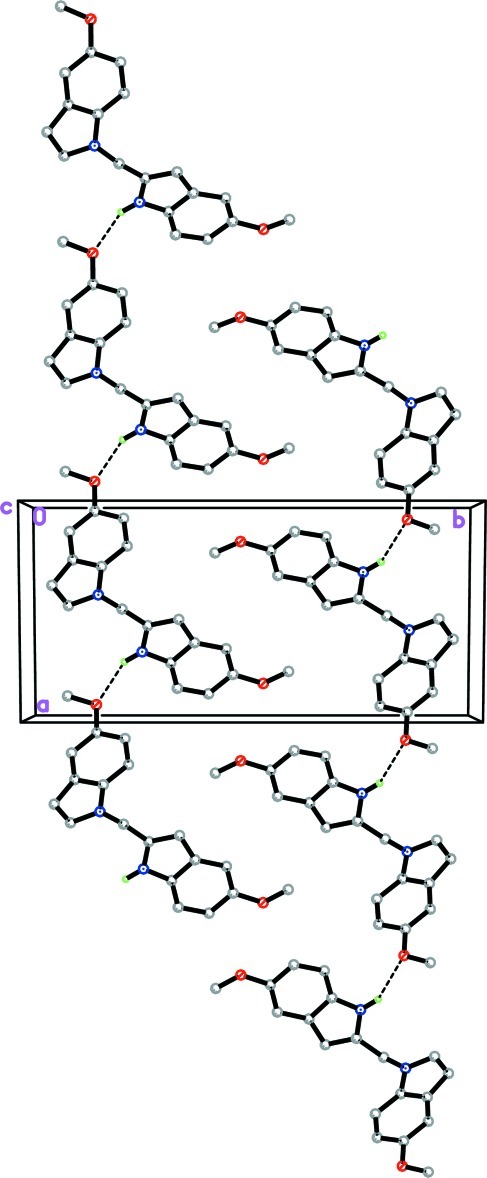
A packing diagram of the title compound viewed along the c axis. For the sake of clarity, H atoms not involved in the intermolecular interactions (dashed lines) have been omitted.

## supplementary crystallographic information

### Comment

Melatonin (*N*-acetyl-5-methoxytryptamine, MLT) is primarily produced by the pineal gland in the brain with a marked circadian rhythm normally peaking in the dark to regulate sleep. MLT acts through activation of two G-protein-coupled receptors, designated as MT_1_ and MT_2_ (Csernus & Mess, 2003). In addition, a low-affinity putative MLT binding site called MT_3_ has been recently characterized as a melatonin-sensitive form of the human enzyme quinine reductase 2 (Nosjean *et al.*, 2000). MLT has found widespread use in the treatment of sleep disorders. Other effects described in the literature include its anti-inflammatory, pain modulatory, antitumor, and antioxidant properties (Blask *et al.*, 2002; Genovese *et al.*, 2005; Mills *et al.*, 2005; Peres, 2005; Sofic *et al.*, 2005; Witt-Enderby *et al.*, 2006). The title compound is an intermediate which could yield, *via* the reported procedure (Attia *et al.*, 2008), various MLT analogues which can be evaluated for their potency and selectivity for MLT receptor subtypes.

In the title compound (Fig. 1), the indole ring systems (N1/C10–C17 & N2/C1–C8) are essentially planar with maximum deviations of 0.015 (2) Å at atom C10 and C2, respectively. In addition, the indole ring systems are almost perpendicular to each other with dihedral angle of 72.17 (7)°. Bond lengths and angles are within the normal range and are comparable to those in the related structures (Narayanan *et al.*, 2011; Deng *et al.*, 2011).

The crystal structure is shown in Fig. 2. The molecules are linked into one dimensional zigzag chains along *a*-axis *via* N2—H1N2···O1 interactions (Table 1).

### Experimental

(5-Methoxy-1*H*-indol-1-yl)(5-methoxy-1*H*-indol-2-yl)methanone (0.50 g, 156.03 mmol) was dissolved in dry THF (5 ml) and was added drop-wise to a cooled (0 °C) suspension of LiAlH_4_/AlCl_3_ in dry diethyl ether (prepared by a slow addition of AlCl_3_ (0.32 g, 2.41 mmol) to a suspension LiAlH_4_ (0.27 g, 7.13 mmol) in dry diethyl ether (15 ml) at 0 °C). The resulting reaction mixture was stirred at 0 °C for one hour and at room temperature for another one hour. The reaction was quenched by a slow addition of saturated sodium sulfate solution. The solids formed were removed by filtration, washed with chloroform (20 ml) and the combined organic phase was dried (Na_2_SO_4_) and evaporated under reduced pressure. The residue was purified by silica gel chromatography (chloroform/methanol/ammonia, 10.0:1.0:0.1) to produce the title compound as a light red powder which was recrystallized from ethanol to give single crystals (*m.p.* 173–174 °C).

### Refinement

N-bound H atom was located in a difference Fourier map and refined freely [N—H = 0.88 (2) Å]. Other H atoms were positioned geometrically (C—H = 0.93–0.97 Å) and refined using a riding model, with *U*_iso_(H) = 1.2 or 1.5*U*_eq_(C). A rotating group model was applied to the methyl groups.

### Crystal data

**Table:**

C_19_H_18_N_2_O_2_	*F*(000) = 648
*M**_r_* = 306.35	*D*_x_ = 1.286 Mg m^−^^3^
Monoclinic, *P*2_1_/*c*	Cu *K*α radiation, λ = 1.54178 Å
Hall symbol: -P 2ybc	Cell parameters from 919 reflections
*a* = 9.4446 (5) Å	θ = 4.5–60.8°
*b* = 19.5625 (8) Å	µ = 0.68 mm^−^^1^
*c* = 8.6657 (5) Å	*T* = 296 K
β = 98.903 (4)°	Plate, pink
*V* = 1581.78 (14) Å^3^	0.92 × 0.20 × 0.06 mm
*Z* = 4	

### Data collection

**Table:**

Bruker APEXII CCD diffractometer	2584 independent reflections
Radiation source: fine-focus sealed tube	2087 reflections with *I* > 2σ(*I*)
Graphite monochromator	*R*_int_ = 0.041
φ and ω scans	θ_max_ = 65.0°, θ_min_ = 4.5°
Absorption correction: multi-scan (*SADABS*; Bruker, 2009)	*h* = −11→11
*T*_min_ = 0.575, *T*_max_ = 0.961	*k* = −22→22
9421 measured reflections	*l* = −8→9

### Refinement

**Table:**

Refinement on *F*^2^	Secondary atom site location: difference Fourier map
Least-squares matrix: full	Hydrogen site location: inferred from neighbouring sites
*R*[*F*^2^ > 2σ(*F*^2^)] = 0.046	H atoms treated by a mixture of independent and constrained refinement
*wR*(*F*^2^) = 0.133	*w* = 1/[σ^2^(*F*_o_^2^) + (0.0478*P*)^2^ + 0.3465*P*] where *P* = (*F*_o_^2^ + 2*F*_c_^2^)/3
*S* = 1.06	(Δ/σ)_max_ < 0.001
2584 reflections	Δρ_max_ = 0.15 e Å^−^^3^
215 parameters	Δρ_min_ = −0.14 e Å^−^^3^
0 restraints	Extinction correction: *SHELXL*, Fc^*^=kFc[1+0.001xFc^2^λ^3^/sin(2θ)]^-1/4^
Primary atom site location: structure-invariant direct methods	Extinction coefficient: 0.0029 (5)

### Special details

**Table:**

Geometry. All e.s.d.'s (except the e.s.d. in the dihedral angle between two l.s. planes) are estimated using the full covariance matrix. The cell e.s.d.'s are taken into account individually in the estimation of e.s.d.'s in distances, angles and torsion angles; correlations between e.s.d.'s in cell parameters are only used when they are defined by crystal symmetry. An approximate (isotropic) treatment of cell e.s.d.'s is used for estimating e.s.d.'s involving l.s. planes.
Refinement. Refinement of *F*^2^ against ALL reflections. The weighted *R*-factor *wR* and goodness of fit *S* are based on *F*^2^, conventional *R*-factors *R* are based on *F*, with *F* set to zero for negative *F*^2^. The threshold expression of *F*^2^ > σ(*F*^2^) is used only for calculating *R*-factors(gt) *etc*. and is not relevant to the choice of reflections for refinement. *R*-factors based on *F*^2^ are statistically about twice as large as those based on *F*, and *R*- factors based on ALL data will be even larger.

### Fractional atomic coordinates and isotropic or equivalent isotropic displacement parameters (Å^2^)

**Table:**

	*x*	*y*	*z*	*U*_iso_*/*U*_eq_	
N1	0.57956 (17)	0.66569 (8)	0.6878 (2)	0.0604 (4)	
N2	0.32036 (18)	0.76234 (9)	0.5805 (2)	0.0588 (4)	
O1	1.08253 (15)	0.66541 (8)	0.44352 (19)	0.0727 (5)	
O2	0.1802 (2)	1.02435 (9)	0.3902 (2)	0.0978 (6)	
C1	0.4392 (2)	0.77296 (11)	0.6916 (2)	0.0573 (5)	
C2	0.4672 (2)	0.84059 (11)	0.7021 (2)	0.0616 (5)	
H2A	0.5421	0.8610	0.7683	0.074*	
C3	0.36179 (19)	0.87542 (10)	0.5938 (2)	0.0536 (5)	
C4	0.3345 (2)	0.94428 (11)	0.5531 (3)	0.0644 (6)	
H4A	0.3924	0.9789	0.6018	0.077*	
C5	0.2203 (2)	0.95918 (11)	0.4396 (3)	0.0675 (6)	
C6	0.1345 (2)	0.90736 (12)	0.3641 (3)	0.0703 (6)	
H6A	0.0593	0.9188	0.2859	0.084*	
C7	0.1585 (2)	0.84013 (12)	0.4025 (3)	0.0661 (6)	
H7A	0.1008	0.8059	0.3519	0.079*	
C8	0.27154 (19)	0.82467 (9)	0.5191 (2)	0.0529 (5)	
C9	0.5147 (2)	0.71548 (12)	0.7821 (3)	0.0709 (6)	
H9A	0.5893	0.7342	0.8602	0.085*	
H9B	0.4470	0.6918	0.8368	0.085*	
C10	0.5303 (2)	0.60163 (11)	0.6481 (3)	0.0728 (6)	
H10A	0.4461	0.5831	0.6732	0.087*	
C11	0.6216 (2)	0.56865 (11)	0.5667 (3)	0.0704 (6)	
H11A	0.6112	0.5244	0.5271	0.084*	
C12	0.73578 (19)	0.61433 (9)	0.5536 (2)	0.0535 (5)	
C13	0.86132 (19)	0.60976 (9)	0.4852 (2)	0.0552 (5)	
H13A	0.8813	0.5708	0.4311	0.066*	
C14	0.95290 (19)	0.66412 (10)	0.5005 (2)	0.0538 (5)	
C15	0.9232 (2)	0.72402 (10)	0.5783 (2)	0.0565 (5)	
H15A	0.9874	0.7603	0.5852	0.068*	
C16	0.8003 (2)	0.72991 (9)	0.6444 (2)	0.0548 (5)	
H16A	0.7802	0.7696	0.6961	0.066*	
C17	0.70772 (19)	0.67448 (9)	0.6312 (2)	0.0505 (4)	
C18	1.1240 (3)	0.60545 (15)	0.3717 (4)	0.0934 (8)	
H18A	1.2164	0.6122	0.3411	0.140*	
H18B	1.0550	0.5953	0.2811	0.140*	
H18C	1.1287	0.5680	0.4440	0.140*	
C19	0.2324 (3)	1.07922 (13)	0.4857 (4)	0.1022 (9)	
H19A	0.1819	1.1201	0.4492	0.153*	
H19C	0.3328	1.0851	0.4823	0.153*	
H19D	0.2184	1.0701	0.5911	0.153*	
H1N2	0.274 (3)	0.7238 (12)	0.559 (3)	0.072 (7)*	

### Atomic displacement parameters (Å^2^)

**Table:**

	*U*^11^	*U*^22^	*U*^33^	*U*^12^	*U*^13^	*U*^23^
N1	0.0524 (9)	0.0644 (10)	0.0664 (11)	0.0041 (7)	0.0159 (7)	0.0093 (8)
N2	0.0544 (9)	0.0598 (10)	0.0637 (11)	−0.0052 (8)	0.0135 (7)	0.0005 (8)
O1	0.0617 (8)	0.0756 (10)	0.0863 (11)	−0.0066 (7)	0.0287 (7)	−0.0043 (8)
O2	0.1246 (15)	0.0672 (10)	0.0933 (14)	0.0037 (9)	−0.0091 (11)	0.0129 (9)
C1	0.0501 (10)	0.0742 (13)	0.0501 (12)	0.0041 (9)	0.0153 (8)	−0.0015 (9)
C2	0.0528 (11)	0.0777 (14)	0.0531 (12)	−0.0001 (9)	0.0040 (8)	−0.0115 (9)
C3	0.0514 (10)	0.0635 (11)	0.0475 (11)	−0.0020 (8)	0.0132 (8)	−0.0084 (8)
C4	0.0669 (12)	0.0638 (13)	0.0615 (13)	−0.0084 (10)	0.0073 (10)	−0.0102 (9)
C5	0.0758 (13)	0.0634 (12)	0.0624 (14)	0.0007 (10)	0.0078 (10)	0.0034 (10)
C6	0.0682 (13)	0.0769 (14)	0.0617 (14)	−0.0004 (11)	−0.0031 (10)	0.0038 (10)
C7	0.0601 (12)	0.0718 (14)	0.0634 (13)	−0.0117 (10)	0.0005 (10)	−0.0038 (10)
C8	0.0495 (10)	0.0603 (11)	0.0513 (11)	−0.0038 (8)	0.0147 (8)	−0.0033 (8)
C9	0.0676 (13)	0.0903 (16)	0.0584 (14)	0.0158 (11)	0.0217 (10)	0.0089 (11)
C10	0.0542 (11)	0.0711 (14)	0.0938 (18)	−0.0107 (10)	0.0132 (11)	0.0141 (12)
C11	0.0587 (12)	0.0594 (12)	0.0917 (17)	−0.0093 (10)	0.0079 (11)	−0.0037 (11)
C12	0.0482 (10)	0.0538 (10)	0.0563 (12)	−0.0028 (8)	0.0015 (8)	0.0027 (8)
C13	0.0543 (10)	0.0534 (10)	0.0569 (12)	0.0020 (8)	0.0061 (8)	−0.0050 (8)
C14	0.0486 (10)	0.0600 (11)	0.0534 (11)	−0.0017 (8)	0.0097 (8)	0.0042 (8)
C15	0.0546 (10)	0.0527 (11)	0.0618 (13)	−0.0058 (8)	0.0078 (8)	0.0018 (8)
C16	0.0592 (11)	0.0502 (10)	0.0538 (12)	0.0011 (8)	0.0053 (8)	−0.0016 (8)
C17	0.0479 (9)	0.0530 (10)	0.0502 (10)	0.0031 (8)	0.0064 (7)	0.0072 (8)
C18	0.0780 (16)	0.1011 (19)	0.110 (2)	0.0006 (14)	0.0436 (14)	−0.0225 (15)
C19	0.112 (2)	0.0623 (15)	0.128 (3)	0.0008 (14)	0.0042 (18)	0.0030 (15)

### Geometric parameters (Å, º)

**Table:**

N1—C10	1.362 (3)	C7—H7A	0.9300
N1—C17	1.385 (2)	C9—H9A	0.9700
N1—C9	1.465 (3)	C9—H9B	0.9700
N2—C1	1.377 (3)	C10—C11	1.359 (3)
N2—C8	1.381 (3)	C10—H10A	0.9300
N2—H1N2	0.88 (2)	C11—C12	1.419 (3)
O1—C14	1.390 (2)	C11—H11A	0.9300
O1—C18	1.411 (3)	C12—C17	1.401 (3)
O2—C5	1.379 (3)	C12—C13	1.408 (3)
O2—C19	1.398 (3)	C13—C14	1.364 (3)
C1—C2	1.349 (3)	C13—H13A	0.9300
C1—C9	1.488 (3)	C14—C15	1.402 (3)
C2—C3	1.431 (3)	C15—C16	1.376 (3)
C2—H2A	0.9300	C15—H15A	0.9300
C3—C8	1.400 (3)	C16—C17	1.387 (3)
C3—C4	1.406 (3)	C16—H16A	0.9300
C4—C5	1.374 (3)	C18—H18A	0.9600
C4—H4A	0.9300	C18—H18B	0.9600
C5—C6	1.396 (3)	C18—H18C	0.9600
C6—C7	1.367 (3)	C19—H19A	0.9600
C6—H6A	0.9300	C19—H19C	0.9600
C7—C8	1.385 (3)	C19—H19D	0.9600
			
C10—N1—C17	107.95 (17)	H9A—C9—H9B	107.6
C10—N1—C9	126.61 (18)	C11—C10—N1	110.38 (18)
C17—N1—C9	125.33 (17)	C11—C10—H10A	124.8
C1—N2—C8	108.88 (17)	N1—C10—H10A	124.8
C1—N2—H1N2	127.1 (16)	C10—C11—C12	107.10 (19)
C8—N2—H1N2	123.6 (16)	C10—C11—H11A	126.5
C14—O1—C18	117.49 (17)	C12—C11—H11A	126.5
C5—O2—C19	118.1 (2)	C17—C12—C13	119.31 (16)
C2—C1—N2	108.97 (18)	C17—C12—C11	106.74 (18)
C2—C1—C9	129.2 (2)	C13—C12—C11	133.94 (19)
N2—C1—C9	121.79 (19)	C14—C13—C12	118.18 (17)
C1—C2—C3	108.28 (17)	C14—C13—H13A	120.9
C1—C2—H2A	125.9	C12—C13—H13A	120.9
C3—C2—H2A	125.9	C13—C14—O1	124.04 (18)
C8—C3—C4	119.24 (18)	C13—C14—C15	121.81 (18)
C8—C3—C2	106.12 (17)	O1—C14—C15	114.14 (16)
C4—C3—C2	134.64 (18)	C16—C15—C14	120.99 (17)
C5—C4—C3	118.42 (19)	C16—C15—H15A	119.5
C5—C4—H4A	120.8	C14—C15—H15A	119.5
C3—C4—H4A	120.8	C15—C16—C17	117.50 (17)
C4—C5—O2	124.4 (2)	C15—C16—H16A	121.2
C4—C5—C6	121.1 (2)	C17—C16—H16A	121.2
O2—C5—C6	114.5 (2)	N1—C17—C16	129.98 (18)
C7—C6—C5	121.5 (2)	N1—C17—C12	107.83 (16)
C7—C6—H6A	119.3	C16—C17—C12	122.19 (18)
C5—C6—H6A	119.3	O1—C18—H18A	109.5
C6—C7—C8	117.88 (19)	O1—C18—H18B	109.5
C6—C7—H7A	121.1	H18A—C18—H18B	109.5
C8—C7—H7A	121.1	O1—C18—H18C	109.5
N2—C8—C7	130.36 (18)	H18A—C18—H18C	109.5
N2—C8—C3	107.76 (17)	H18B—C18—H18C	109.5
C7—C8—C3	121.86 (19)	O2—C19—H19A	109.5
N1—C9—C1	114.58 (18)	O2—C19—H19C	109.5
N1—C9—H9A	108.6	H19A—C19—H19C	109.5
C1—C9—H9A	108.6	O2—C19—H19D	109.5
N1—C9—H9B	108.6	H19A—C19—H19D	109.5
C1—C9—H9B	108.6	H19C—C19—H19D	109.5
			
C8—N2—C1—C2	−0.1 (2)	N2—C1—C9—N1	63.6 (3)
C8—N2—C1—C9	178.52 (17)	C17—N1—C10—C11	−0.4 (2)
N2—C1—C2—C3	0.3 (2)	C9—N1—C10—C11	−176.8 (2)
C9—C1—C2—C3	−178.2 (2)	N1—C10—C11—C12	0.0 (3)
C1—C2—C3—C8	−0.3 (2)	C10—C11—C12—C17	0.5 (2)
C1—C2—C3—C4	179.3 (2)	C10—C11—C12—C13	179.3 (2)
C8—C3—C4—C5	−0.7 (3)	C17—C12—C13—C14	1.4 (3)
C2—C3—C4—C5	179.7 (2)	C11—C12—C13—C14	−177.2 (2)
C3—C4—C5—O2	179.0 (2)	C12—C13—C14—O1	177.71 (17)
C3—C4—C5—C6	−1.1 (3)	C12—C13—C14—C15	−1.6 (3)
C19—O2—C5—C4	−17.5 (4)	C18—O1—C14—C13	−2.9 (3)
C19—O2—C5—C6	162.6 (2)	C18—O1—C14—C15	176.4 (2)
C4—C5—C6—C7	1.6 (4)	C13—C14—C15—C16	0.9 (3)
O2—C5—C6—C7	−178.5 (2)	O1—C14—C15—C16	−178.47 (17)
C5—C6—C7—C8	−0.2 (3)	C14—C15—C16—C17	0.0 (3)
C1—N2—C8—C7	178.2 (2)	C10—N1—C17—C16	−178.4 (2)
C1—N2—C8—C3	−0.1 (2)	C9—N1—C17—C16	−1.9 (3)
C6—C7—C8—N2	−179.7 (2)	C10—N1—C17—C12	0.7 (2)
C6—C7—C8—C3	−1.6 (3)	C9—N1—C17—C12	177.15 (18)
C4—C3—C8—N2	−179.48 (17)	C15—C16—C17—N1	178.80 (18)
C2—C3—C8—N2	0.3 (2)	C15—C16—C17—C12	−0.2 (3)
C4—C3—C8—C7	2.1 (3)	C13—C12—C17—N1	−179.73 (16)
C2—C3—C8—C7	−178.18 (18)	C11—C12—C17—N1	−0.7 (2)
C10—N1—C9—C1	−105.6 (2)	C13—C12—C17—C16	−0.5 (3)
C17—N1—C9—C1	78.6 (3)	C11—C12—C17—C16	178.44 (17)
C2—C1—C9—N1	−118.1 (2)		

### Hydrogen-bond geometry (Å, º)

**Table:**

*D*—H···*A*	*D*—H	H···*A*	*D*···*A*	*D*—H···*A*
N2—H1*N*2···O1^i^	0.88 (2)	2.24 (3)	3.037 (2)	151 (2)

Symmetry code: (i) *x*−1, *y*, *z*.
